# Anesthesia risks should not be ignored beyond complications from pulmonary surgery after SARS-Cov-2 infection

**DOI:** 10.1097/JS9.0000000000001570

**Published:** 2024-05-08

**Authors:** Kai Kang, Ailin Zhao, Yijun Wu

**Affiliations:** a Division of Thoracic Tumor Multimodality Treatment, Cancer Center, West China Hospital, Sichuan University; b Department of Hematology, West China Hospital, Sichuan University; c Laboratory of Clinical Cell Therapy, West China Hospital, Sichuan University, Chengdu, Sichuan, People’s Republic of China

*Dear Editor,*

Since the outbreak of the SARS-CoV-2 (severe acute respiratory syndrome coronavirus 2), the global healthcare systems have been subjected to severe challenges. Currently, a series of issues have become focal points of mutual concern for both medical professionals and normal people that involve preoperative assessment, timing of surgery, prevention of postoperative complications and rehabilitation for patients after SARS-CoV-2 infection. We were recently intrigued by the study conducted by Duan *et al*.^1^ regarding the optimal timing for pulmonary resection surgery after SARS-CoV-2 infection, which has filled a gap in this knowledge. The authors employed a multicenter retrospective cohort design, encompassing a total of 400 patients, 80.5% of whom were diagnosed with lung cancer by postoperative pathological examination. Their main findings indicated that, as the time interval between SARS-CoV-2 infection and pulmonary surgery increased, the incidence of postoperative complications gradually decreased, notably with the significantly higher risk of greater than grade II complications in the 2-week or less and 2-week to 4-week groups compared to the greater than 4-week group, suggesting that lung resection surgery should be delayed for at least 4 weeks post-SARS-CoV-2 infection. Limitations of the study include potential information bias due to its retrospective design and the possible influence of unrecorded factors. Furthermore, the study scope was confined to patients within a short timeframe, and the impact of long-term follow-up and outcomes remains unclear. This study holds crucial clinical significance by providing physicians with a reference for the timing of pulmonary resection surgery in SARS-CoV-2-infected patients, aiding in reducing the incidence of postoperative complications and improving surgical success rates. Larger prospective studies are needed in the future to validate these findings and further explore the impact of timing on long-term patient prognosis.

However, in addition to complications related to lung surgery, consideration should also be given to the impact of SARS-CoV-2 infection on other factors such as the anesthesia risk. Findings from an international multicenter prospective clinical trial suggest that for selective surgery, patients undergoing surgery within 0–2 weeks, 3–4 weeks, and 5–6 weeks after the diagnosis of SARS-CoV-2 infection had higher mortality rates compared to the uninfected, with rates of 4.1% (95% CI 3.3–4.8%), 3.9% (95% CI 2.6–5.1%), and 3.6% (95% CI 2.0–5.2%), respectively; whereas patients undergoing surgery at least 7 weeks after diagnosis had a similar 30-day mortality rate of 1.5% (95% CI 0.9–2.1%) to uninfected patients^2^. Therefore, we hypothesize that solely focusing on surgical complications is insufficient and suggest that the delay should be extended beyond 4 weeks after SARS-CoV-2 infection. We believe that much more factors should be taken into account to comprehensively assess the timing of surgery following SARS-CoV-2 infection in patients, especially the anesthesia risk.

In detail, lung surgery heavily relies on pulmonary function, which is specially and easily impaired by SARS-CoV-2 infection. Consequently, the risk of surgical anesthesia after SARS-CoV-2 infection can be significantly increased, including: *difficulty in endotracheal intubation*: SARS-CoV-2 infection causes inflammation of the upper respiratory tract, leading to mucosal congestion and edema, particularly in special presentations such as laryngeal congestion and epiglottitis with a risk of postoperative airway obstruction; *airway hyperreactivity*: SARS-CoV-2 invasion of the upper respiratory tract triggers inflammation and high sensitivity of the airway, where the use of certain anesthetic drugs and airway procedures may induce local irritation leading to airway spasm, presenting with laryngospasm, bronchospasm, airway narrowing, sudden increase in airway resistance and expiratory respiratory distress, with severe cases causing hypoxia and CO_2_ retention; *ventilation insufficiency and hypoxemia*: SARS-CoV-2 can invade the lower respiratory tract, causing lung inflammation and consolidation that result in the decreased lung ventilation and gas exchange function, predisposing to hypoxemia; *cardiovascular risks*: SARS-CoV-2 invasion of the cardiovascular system may induce myocarditis, myocardial injury, broken heart syndrome, arrhythmias, pulmonary hypertension, increasing the risk of imbalance in myocardial oxygen supply and hemodynamic changes; *immune suppression*: anesthetic drugs can suppress the body’s immune function, which may prolong the replication time of active viruses in the body, slowing down the recovery after SARS-CoV-2 infection; *coagulation dysfunction*: SARS-CoV-2 infection can affect the body’s coagulation function, significantly increasing the likelihood of stroke and pulmonary embolism.

Therefore, we recommend a comprehensive and individualized preoperative assessment when considering multiple factors such as the patient’s underlying conditions, the severity of symptoms and surgical anesthesia risks for patients to select the optimal timing for pulmonary surgery after SARS-CoV-2 infection, while the postoperative complications are just one of these considerations.

## Ethics approval

Not applicable.

## Consent

Not applicable.

## Sources of funding

This work was supported by the Postdoctoral Fellowship Program of CPSF (No. GZB20230481), National Natural Science Foundation of China (No. 82303773, No. 82303772, and No. 82303694), Natural Science Foundation of Sichuan Province (No. 2023NSFSC1885), and Key Research and Development Program of Sichuan Province (No. 23ZDYF2836).

## Author contribution

Y.W., K.K., and A.Z.: writing the paper; Y.W.: study concept and review and editing.

## Conflicts of interest disclosure

The author declares no conflict of interest.

## Research registration unique identifying number (UIN)

Not applicable.

## Guarantor

Dr Yijun Wu.

## Data availability statement

Not applicable.

## Provenance and peer review

Not applicable.
